# Intraoperative hemoadsorption in high-risk patients with infective endocarditis

**DOI:** 10.1371/journal.pone.0266820

**Published:** 2022-07-28

**Authors:** Zaki Haidari, Ender Demircioglu, Kristina Boss, Bartosz Tyczynski, Matthias Thielmann, Bastian Schmack, Andreas Kribben, Alexander Weymann, Mohamed El Gabry, Arjang Ruhparwar, Daniel Wendt

**Affiliations:** 1 Department of Thoracic and Cardiovascular Surgery, West German Heart and Vascular Center Essen, Essen, Germany; 2 Department of Nephrology, University Hospital Essen, Essen, Germany; Policlinico S. Orsola-Malpighi, ITALY

## Abstract

**Background:**

Postoperative sepsis is an important cause of morbidity and mortality in patients with infective endocarditis undergoing surgical therapy. Blood purification using hemoadsorption therapy shows promising results in the treatment of sepsis. In this study, the clinical effects of intraoperative hemoadsorption in high-risk patients with infective endocarditis were evaluated.

**Methods:**

Eligible candidates were high-risk patients with infective endocarditis undergoing cardiac surgery between January 2014 and December 2019. Patients with intraoperative hemoadsorption (hemoadsorption) were compared to patients without hemoadsorption (control). The endpoints were the incidence of postoperative sepsis, sepsis-associated death and in-hospital mortality. Additionally, postoperative vasopressor need, systemic vascular resistance indices and Sequential Organ Failure Assessment (SOFA) scores were compared.

**Results:**

After propensity score matching, 70 high-risk patients were included. Postoperative sepsis occurred in 14 patients in the hemoadsorption group and in 16 patients in the control group, *p* = 0.629. Four patients died due to postoperative sepsis in the hemoadsorption group, while 11 postoperative septic patients died in the control group, *p* = 0.041. In-hospital mortality was 34% in the hemoadsorption group versus 43% in the control group, *p* = 0.461. On ICU-admission and the first postoperative day, the cumulative vasopressor need was 0.17 versus 0.25 μg/kgBW/min, *p* = 0.123 and 0.06 versus 0.11 μg/kgBW/min, *p* = 0.037, and the systemic vascular resistance index was 1448 versus 941 dyn·s·cm^-5^, *p* = 0.013 and 1156 versus 858 dyn·s·cm^-5^, *p* = 0.110 in the hemoadsorption versus control group, respectively. Postoperative course of SOFA score normalized significantly (*p* = 0.01) faster in the hemoadsorption group.

**Conclusions:**

In high-risk cardiac surgical patients with infective endocarditis, intraoperative hemoadsorption significantly reduced sepsis-associated mortality. Furthermore, intraoperative hemoadsorption resulted in significant faster recovery of hemodynamics and organ function. Intraoperative hemoadsorption seems to attenuate the severity of postoperative sepsis.

## Introduction

Postoperative sepsis and subsequent organ failure is an important cause of mortality in patients undergoing cardiac surgery for infective endocarditis [1, 2]. Systemic inflammatory response in combination with systemic infection resulting in severe sepsis is orchestrated with the cytokines as messengers [3–12]. We previously reported that intraoperative hemoadsorption reduced the incidence of postoperative sepsis and sepsis-related death in patients with infective endocarditis of the native mitral valve undergoing surgical therapy [13]. Furthermore, we found that European System for Cardiac Operative Risk Evaluation (EuroSCORE) II was the only independent predictor of sepsis-related mortality. However, this novel adjunctive therapy concept requires further investigation in terms of patient selection and timing of application. In this study, we aimed to evaluate the clinical effects of intraoperative hemoadsorption in high-risk patients with infective endocarditis undergoing cardiac surgery.

## Methods

### Patients

Eligible participants for this retrospective study were patients with definitive infective endocarditis undergoing surgical therapy with cardiopulmonary bypass from January 2014 through to December 2019. The modified Duke criteria were followed to verify definite infective endocarditis [14]. Exclusion criteria were cardiac-device related infective endocarditis, requiring simple retraction without cardiopulmonary bypass. For every patient, the EuroSCORE II was calculated using the online calculator (http://www.euroscore.org/calc.html) and all patients with a EuroSCORE II >8% were selected for this study [15]. The study was reviewed and approved by the institutional ethics committee (19-8743-BO) and all patients provided written informed consent before the operation.

### Operation techniques

Cardiac surgery was performed under general anesthesia and endotracheal intubation. Preoperative transesophageal echocardiography was performed to evaluate cardiac and valvular function. Standard aortic and caval cannulation techniques were applied. Cardioplegic arrest was achieved by crystalloid Bretschneider cardioplegia (Custodiol, Dr. Franz Koehler Chemie, Bensheim, Germany). The addition of intraoperative hemoadsorption was decided by the operating surgeon and guided by multiple factors including preoperative clinical (e.g. fever) and inflammatory status, microbiological profile and perioperative hemodynamics. Patients with fever, high inflammatory parameters, presence of staphylococcal species as causative microorganism and high inotropic support were more likely to receive intraoperative hemoadsorption. In the cases selected for intraoperative hemoadsorption, a hemoadsorption device Cytosorb (Cytosorbents^®^, Monmouth Junction, NJ, USA) was installed in a parallel circuit of the cardiopulmonary bypass (CPB) machine during the surgical procedure.

### Hemoadsorption therapy

**Fig 1:**
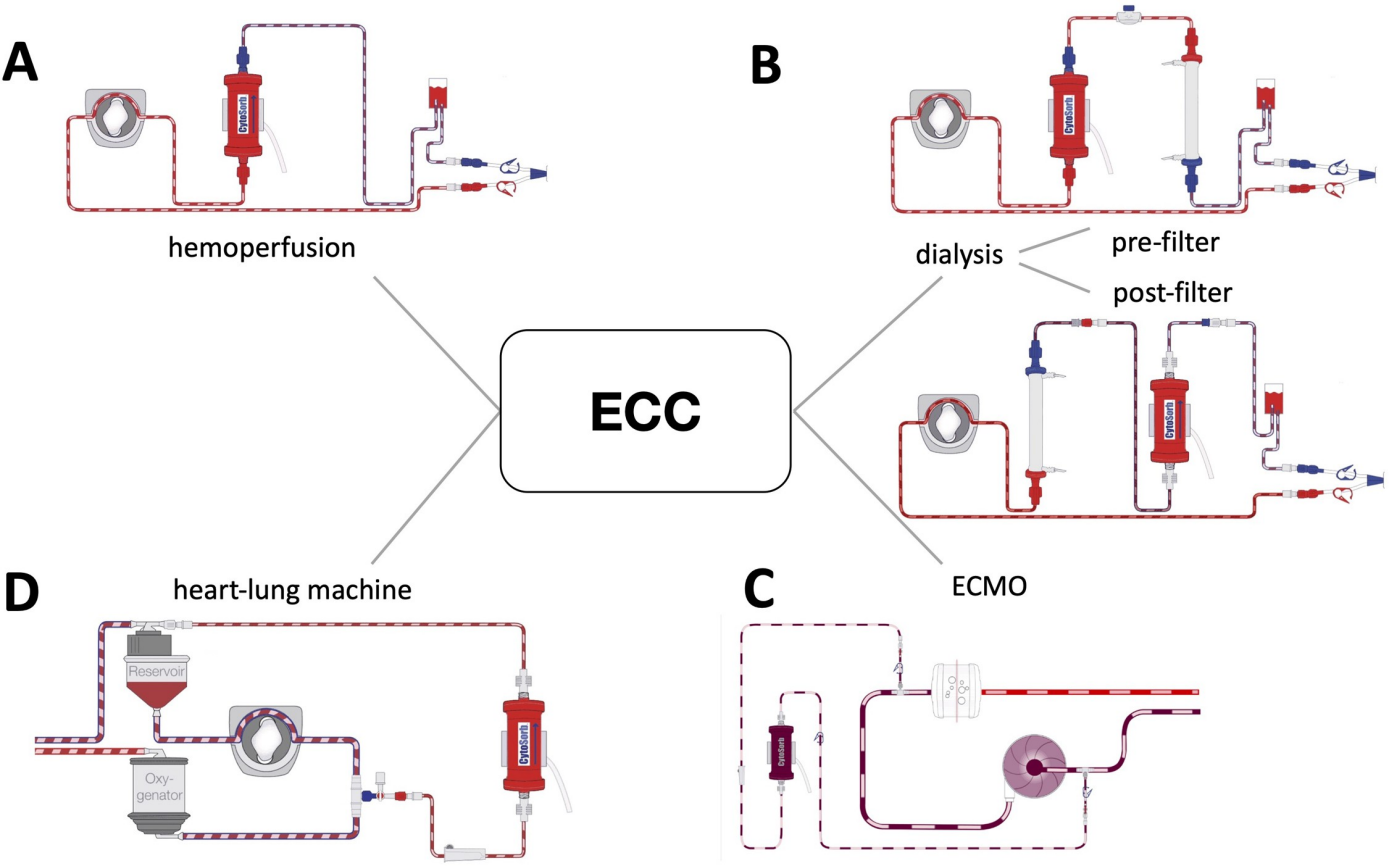
Potential incorporation modes of hemadsorption in various extracorporeal circulation circuits (A: stand-alone, B: continuous renal replacement therapy, C: extracorporeal membrane oxygenation, D: cardiopulmonary bypass). ECC: extracorporeal circulation.

Hemoadsorption therapy with CytoSorb (Cytosorbents^®^, Monmouth Junction, NJ, USA) is based on extracorporeal blood purification that adsorbs excessive levels of inflammatory mediators with the aim to specifically modulate the overshooting immune response, mitigate the cytotoxic effects of elevated cytokine levels and increase the chances for recovery. The cartridge, which can be used as stand-alone or integrated into various extracorporeal circuits, such as continuous renal replacement therapy, CPB and extracorporeal membrane oxygenation (Fig 1), is filled with biocompatible, porous polymer beads covered with a divinylbenzen coating. Each polymer bead is between 300 and 800 μm in size and has pores and channels, giving it a large (40,000 m^2^) effective surface area for binding hydrophobic small and middle molecules.

### Postoperative care

All patients were referred to the cardiac surgical intensive care unit (ICU) with invasive hemodynamic and pulmonary monitoring, and guideline-directed antibiotic and supportive therapy. Hemodynamic monitoring included standard arterial, central venous and pulmonary artery catheter installation for invasive blood pressure and oxygen saturation measurement and cardiac output measurement by transpulmonary thermodilution. Furthermore, systemic vascular resistance index (SVRI) was calculated from data derived from invasive monitoring. Additionally, routinely measured lactate levels were used for the evaluation of the metabolic state. Fluid and vasopressor (primarily epinephrine and norepinephrine) therapy were guided by several factors, including blood pressure, heart rhythm, preoperative cardiac function, SVRI and metabolic parameters such as lactate measured by arterial blood gas analysis. Standard laboratory analysis included daily white blood counts (WBC), C-reactive protein (CRP) concentration and procalcitonin (PCT) measurement.

### Outcome measures

The primary endpoints of the study were the incidence of postoperative sepsis as defined by the third international consensus definitions for sepsis and septic shock, sepsis-related death and in-hospital mortality. In short, postoperative sepsis was defined as occurrence of new organ dysfunction. Septic shock was defined by a vasopressor requirement to maintain a mean arterial pressure of 65 mm Hg or greater and serum lactate level greater than 2 mmol/L in the absence of hypovolemia. Sepsis-related death was defined as in-hospital mortality due to sepsis and septic shock [16]. Secondary endpoints included postoperative need for mechanical circulatory support, organ failure, ICU- and hospital stay. Additionally, postoperative cumulative need for epinephrine and norepinephrine and SVR indices on ICU-admission and first postoperative day and postoperative course of inflammatory parameters and lactate were evaluated. Furthermore, individual Sequential Organ Failure Assessment (SOFA) Scores for each patient was assessed preoperatively and postoperatively up to seventh postoperative day. The SOFA score was calculated on a scale ranging from 0 to 6 for each organ system, resulting in a total score from 0 to 24 [17] and the investigators (Z.H. and D.W.) were blinded to treatment allocation.

### Statistical analysis

Data was analyzed using SPSS software version 25 (SPSS Inc., Chicago, IL, USA). A logistic regression analysis was performed to calculate the propensity score for the selection of patients for both the hemoadsoprtion and control groups. The variables used included age, left ventricular function, dialysis dependency, previous cardiac surgery, presence of prosthetic valve infective endocarditis, days between diagnosis and surgery, positive blood culture for staphylococcal species and application of postoperative hemoadsorption. Matching was performed using 1:1 nearest neighbor with a matching tolerance of 0.1 in the overall propensity score. Continuous variables were expressed as mean and median with standard deviation (SD) and interquartile range (IQR), respectively, and compared using Student’s t-test or the Mann-Whitney test. Categorical data were expressed as number of patients and frequencies, and compared using the chi-square test. An analysis of variance (ANOVA) was computed for the SOFA score.

## Results

### Baseline characteristics

**Fig 2:**
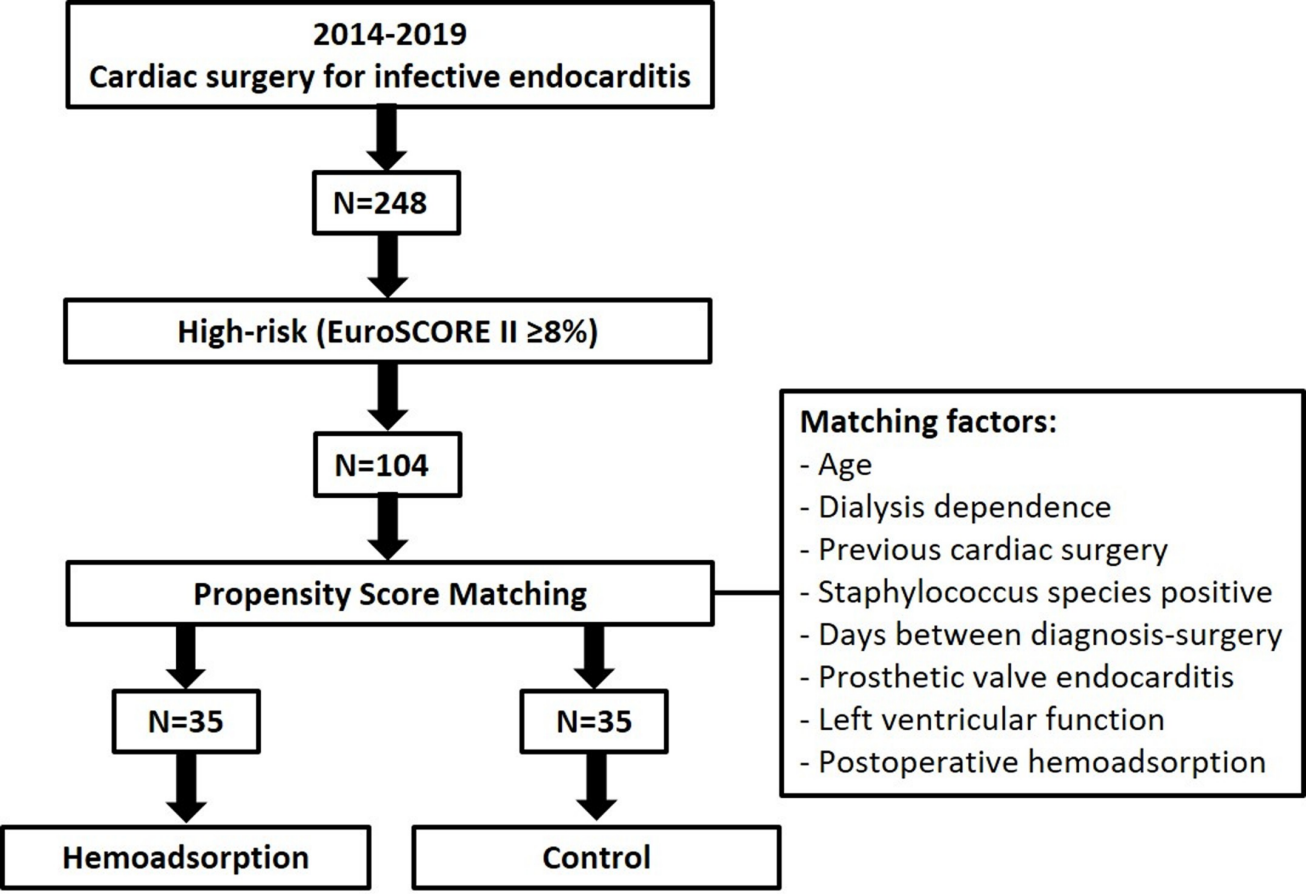
Study flowchart. EuroSCORE: European System for Cardiac Operative Risk Evaluation.

From January 2014 through December 2019, 248 patients underwent cardiac surgery with cardiopulmonary bypass for infective endocarditis. 104 patients were classified as high-risk patients as defined by the EuroSCORE II higher then 8%. After propensity score matching, 70 high-risk patients were included in this study (Fig 2). Preoperative baseline characteristics of the patients are outlined in Table 1. Median age at the time of operation was 73 years (IQR 60–78) and 69% of the patients were male. The median EuroSCORE II for the total population was 15% (IQR 11–31). Median time between definitive diagnosis of infective endocarditis and surgery was 10 (IQR 5–25) days versus 14 days (IQR 3–26), *P* = 0.473 in the hemoadsorption and the control group. Preoperative echocardiography showed preserved left ventricular fraction (LVEF>50%) in 47 patients (67%) and poor (LVEF<35%) left ventricular function in 4 patients (6%). Before induction of anesthesia, 13 patients were intubated and 12 patients needed vasopressor therapy. There were no significant differences in terms of demographics, hemodynamic and pulmonary status, the levels of preoperative inflammatory parameters and SOFA score between the two groups. Concomitant non-endocarditis valve disease requiring surgical therapy was present in 25 patients (5 aortic valve, 9 mitral valve and 11 tricuspid valve). The most commonly identified causative pathogen for infective endocarditis (Table 2) was staphylococcus species in 25 patients of which 20 patients with staphylococcus aureus.

**Table 1:** Baseline characteristics.

Variable	Hemoadsorption	Control	*p*
	N = 35	N = 35	
**Demographics**			
Age, years	70±10	68±13	0.647
Gender, male	24 (69)	24 (69)	1.000
Coronary artery disease	13 (37)	16 (46)	0.467
Pulmonary disease	8 (23)	7 (20)	0.771
Dialysis dependent	3 (9)	2 (6)	0.643
Liver disease	4 (11)	1 (3)	0.164
Peripheral vascular disease	8 (23)	9 (26)	0.780
Previous CABG	7 (20)	7 (20)	1.000
Previous PCI	4 (11)	4 (11)	1.000
Previous valve surgery	17 (49)	18 (37)	0.811
EuroSCORE II	14 (11–30)	19 (11–38)	0.339
**Clinical status**			
NYHA III-IV	21 (60)	17 (49)	0.337
Intubated	6 (17)	7 (20)	0.759
Vasopressor need	6 (17)	6 (17)	1.000
SOFA score	2 (2–4)	2 (1–4)	1.000
**Inflammatory status**			
C-reactive protein, mg/dL	6.4 (3–11)	5.9 (1.08–9.23)	0.814
Procalcitonin, ng/ml	0.21 (0.08–0.62)	0.31 (0.13-1-12)	0.481
White blood count, 10^9^/L	8.9 (6.6–11.7)	8.6 (6.4–12.9)	0.698

Data are presented as mean±SD, median (IQR) or number (%); CABG, coronary artery bypass grafting; PCI, percutaneous coronary intervention; EuroSCORE, European System for Cardiac Operative Risk Evaluation; NYHA, New York Heart Association; SOFA, Sequential Organ Failure Assessment

**Table 2:** Microbiological profile.

Variable	Hemoadsorption	Control	*p*
	N = 35	N = 35	
Staphylococcus species	12 (34)	13 (37)	0.803
Staphylococcus aureus	10 (29)	10 (29)	1.000
Streptococcus species	8 (23)	1 (3)	0.012
Enterococcus species	9 (26)	9 (26)	1.000
Others	2 (6)	3 (9)	0.643
Negative culture	4 (11)	9 (26)	0.124

Data are presented as number (%)

### Operative characteristics

Table 3 represents operative characteristics of the two groups. Infective endocarditis affecting one valve was present in 59 patients, and two or three valves in 10 and one patient, respectively. Prosthetic valve infective endocarditis was present in 43 patients, 22 patients from the hemoadsorption group versus 21 patients in the control group, respectively. Concomitant coronary artery bypass grafting was performed in 13 patients. Cardiopulmonary bypass and aortic cross-clamp times were comparable.

**Table 3:** Operative characteristics.

Variable	Hemoadsorption	Control	*p*
	N = 35	N = 35	
Isolated AV procedure	8 (23)	8 (23)	1.000
Combined AV and MV procedure	5 (14)	3 (9)	0.452
Combined AV and TV procedure	5 (14)	3 (9)	0.452
Combined MV and TV procedure	4 (11)	8 (23)	0.205
Combined AV, MV and TV procedure	2 (6)	3 (9)	0.643
Concomitant CABG	4 (11)	9 (26)	0.124
Cardiopulmonary bypass time, minutes	127 (105–177)	138 (108–195)	0.459
Aortic cross-clamp time, minutes	84 (69–118)	82 (67–121)	0.883

Data are presented as number (%) or median (IQR); AV, aortic valve; MV, mitral valve; TV, tricuspid valve CABG, coronary artery bypass grafting

### Endpoints

The endpoints are summarized in Table 4. Postoperative sepsis occurred in 14 (40%) patients in the hemoadsorption and in 16 patients (46%) in the control group (OR: 0.792, CI: 0.307–2.044, *p* = 0.629). Sepsis-related death occurred in four patients in the hemoadsorption group and in 11 patients in the control group (OR: 0.282, CI: 0.080–0.995, *p* = 0.041). In-hospital mortality was 34% in the hemoadsorption group and 43% in the control group (OR: 0.696, CI: 0.264–1.830, *p* = 0.461). Other causes of in-hospital mortality in the hemoadsorption group were one intraoperative left ventricular posterior wall rupture, one left and one right heart failure, two in-hospital cardiac arrests, three septic shocks due to secondary infection (one candida albicans infection, one unknown, and one deep sternal wound infection, all not related to the index pathogen and to endocarditis). Other causes of death in the control group were one intraoperative left ventricular posterior wall rupture, one cardiogenic shock and two secondary infections (one respiratory failure due to herpes simplex virus pneumonia and one deep sternal wound infection).

**Table 4:** Endpoints.

Variable	Hemoadsorption	Control	*p*
	N = 35	N = 35	
**Primary**			
Sepsis	14 (40)	16 (46)	0.629
Sepsis-associated mortality	4 (11)	11 (31)	0.041
In-hospital mortality	12 (34)	15 (43)	0.461
**Secondary**			
Postoperative IABP/ECMO	4 (11)	5 (14)	0.721
Dialysis	9 (26)	11 (31)	0.649
Reintubation	6 (17)	14 (40)	0.034
ICU-stay, days	7 (4–11)	8 (5–15)	0.228
Hospital stay, days	13 (8–18)	15 (11–20)	0.195

Data are presented as number (%) or median (IQR) IABP, intra-aortic balloon-pump; ECMO, extracorporeal membrane oxygenation; ICU, intensive care unit.

Mechanical circulatory support with extracorporeal membrane oxygenation (ECMO) was necessary in four patients, two in the hemoadsorption group and two in the control group, *p* = 1.000. Intra-aortic balloon pump (IABP) support was applied in six patients, two in the hemoadsorption group and four in the control group, *p* = 0.393. One patient received both ECMO and IABP support. Postoperative new renal failure requiring hemodialysis was present in 20 patients, nine in the hemoadsorption group and 11 in the control group, *p* = 0.649. Respiratory failure requiring reintubation occurred in 20 patients, six in the hemoadsorption group and 14 in the control group, *p* = 0.034. Among all in-hospital deaths (N = 27), 13 patients developed respiratory failure, three in the hemoadsorption group and 10 in the control group, *p* = 0.031. There were no clinical relevant differences in the ICU- and hospital stay between the groups.

**Fig 3:**
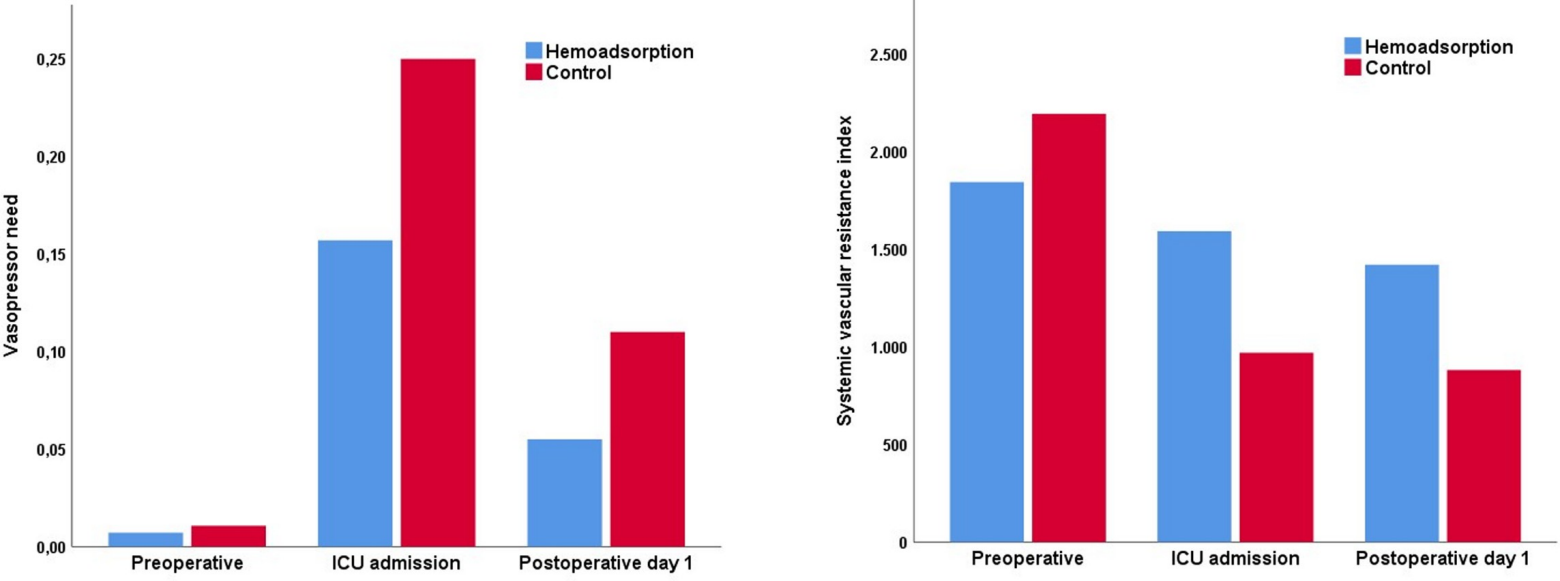
Median vasopressor need in μg/kgBW/min (A) and systemic vascular resistance index in dyn·s·cm^-5^ (B). ICU: intensive care unit.

On ICU-admission, the cumulative need for epinephrine and norepinephrine (Fig 3A) was 0.17 μg/kgBW/min (IQR 0.10–0.30) in the hemoadsorption group and 0.25 μg/kgBW/min (IQR 0.12–0.52) in the control group, *p* = 0.123. On postoperative day 1, the median need for epinephrine and norepinephrine decreased to 0.06 μg/kgBW/min (IQR 0.00–0.13) in the hemoadsorption group and 0.11 μg/kgBW/min (IQR 0.03–0.38) in the control group, *p* = 0.037. Furthermore, an SVRI (Fig 3B) of 1448 dyn·s·cm^-5^ (1133–2065) in the hemoadsorption group versus 941 dyn·s·cm^-5^ (IQR 788–1521) in the control group was observed on ICU-admission, *p* = 0.013. On postoperative day 1, the SVRI had decreased to 1156 dyn·s·cm^-5^ (782–1695) in the hemoadsorption group and in the control group it had decreased to 858 dyn·s·cm^-5^ (715–1340), *p* = 0.110.

**Fig 4:**
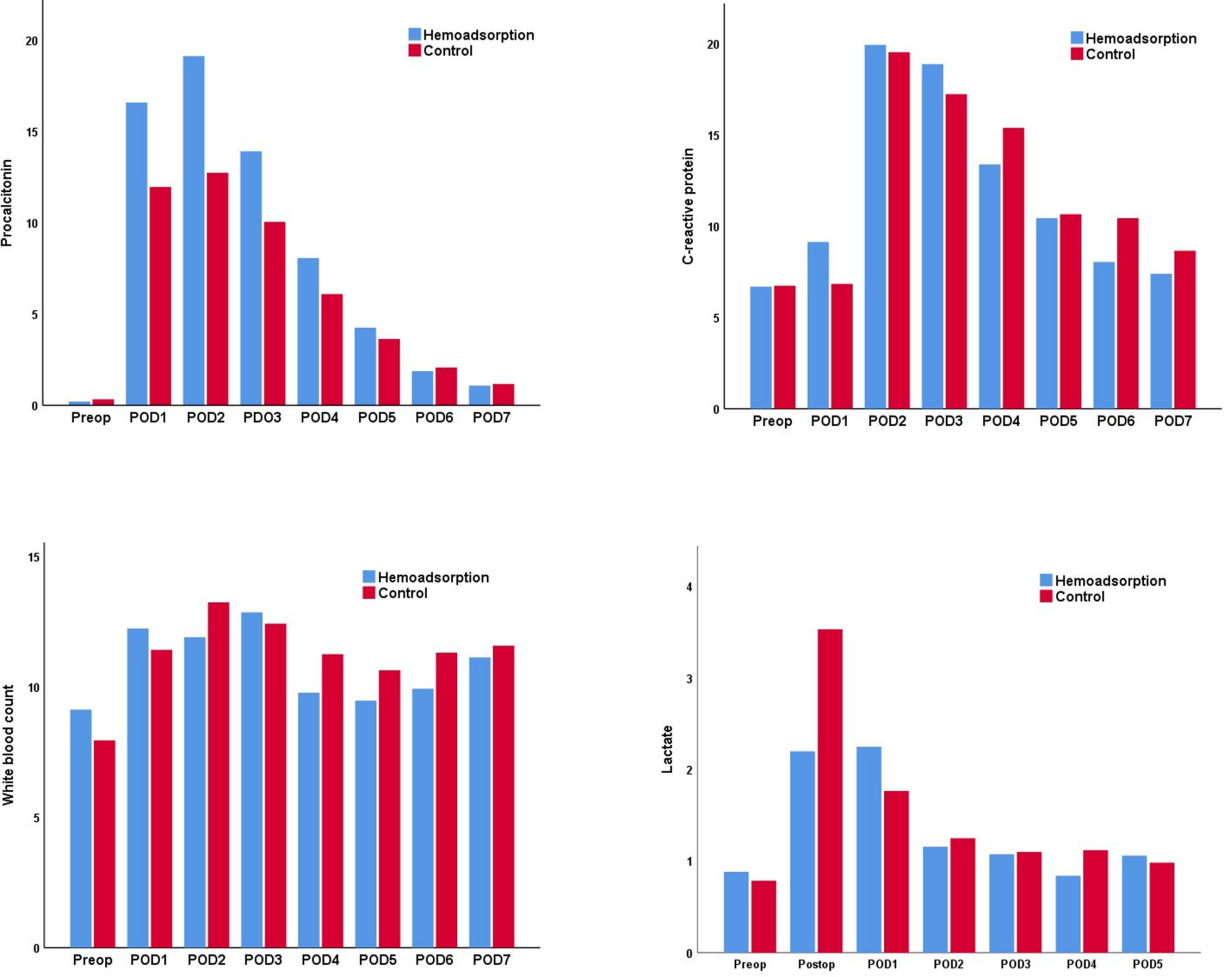
Median levels of procalcitonin in ng/ml (A), C-reactive protein in mg/dl (B), white blood count x1000/ml (C) and lactate in mmol/l (D). POD: postoperative day.

The inflammatory parameters (Fig 4) increased postoperatively in both groups abruptly and peaked on the second postoperative day. PCT levels (Fig 4A) were higher (not statistically significant) in the hemoadsorption group than in the control group. CRP (Fig 4B) and WBC (Fig 4C) showed a comparable course between the two groups with no significant differences. Lactate levels (Fig 4D) on the first postoperative day were lower in the hemoadsorption group compared to the control group, however it did not reach statistical significance.

**Fig 5:**
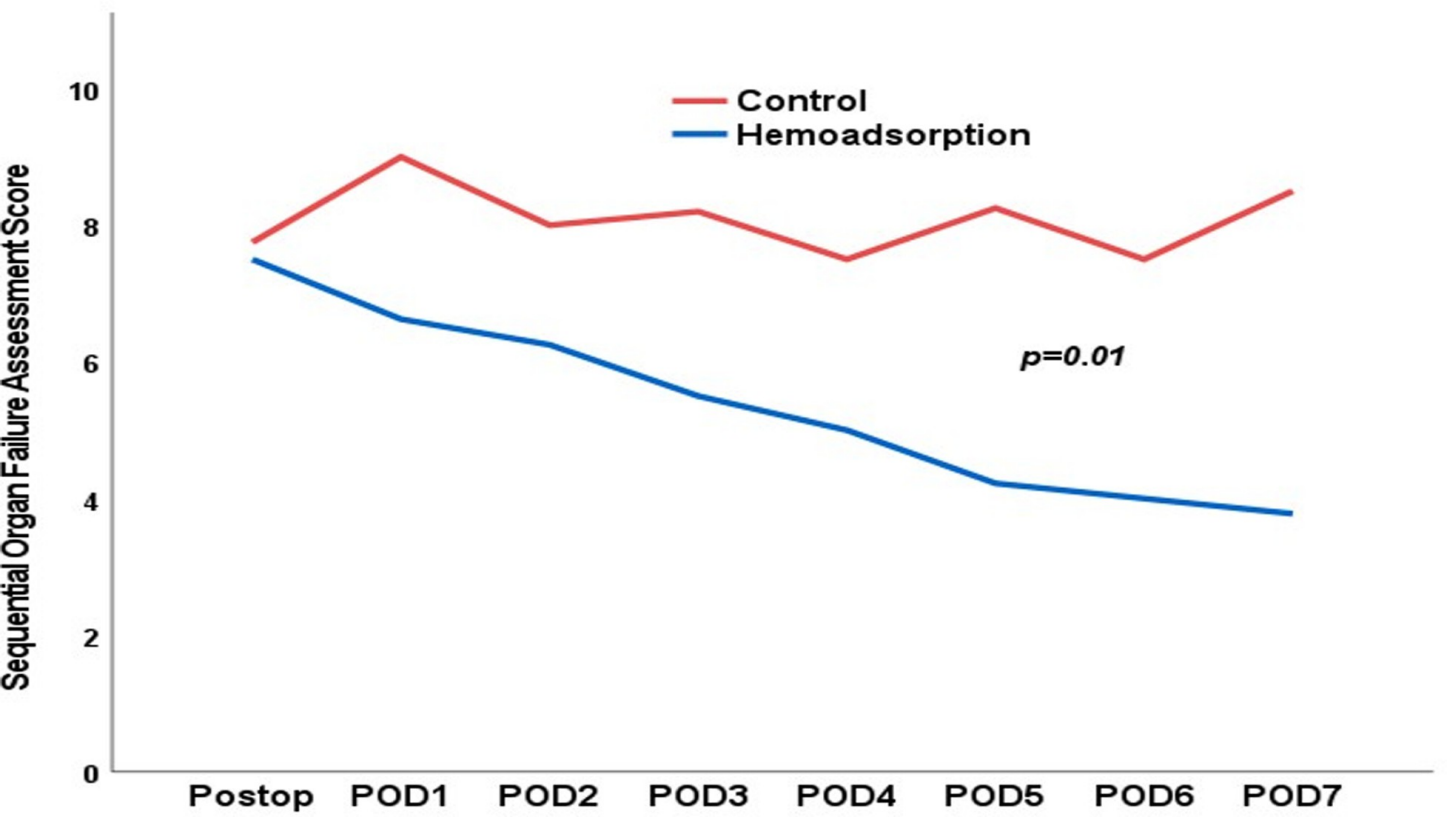
Postoperative course of SOFA score. SOFA: Sequential Organ Failure Assessment.

In regard to the SOFA scores (Fig 5), we observed a significant (*p*<0.001) increase in both groups on the day of operation compared to preoperatively. However, the SOFA score decreased in the hemoadsorption group to preoperative levels in the first seven postoperative days. In the control group, the SOFA score remained high during the first week after the operation. The course of SOFA score was significantly (*p* = 0.01) in favor of the hemoadsorption group.

## Discussion

In the current comparative study, we sought to evaluate the clinical effects of intraoperative hemoadsorption in high-risk patients with infective endocarditis undergoing surgical therapy. Although intraoperative hemoadsorption did not reduce the incidence of postoperative sepsis, sepsis-associated death was significantly lower in patients with intraoperative hemoadsorption therapy than in the control group. Additionally, this difference was reinforced by the favorable hemodynamic parameters and reintubation rates in patients receiving intraoperative hemoadsorption. Furthermore, the SOFA score showed significant faster normalization during the postoperative course in the hemoadsorption group.

Postoperative sepsis remains an important cause of mortality in patients with infective endocarditis undergoing surgical therapy. In our previous report, the use of intraoperative hemoadsorption in patients with native mitral valve infective endocarditis seemed to reduce the incidence of postoperative sepsis and sepsis-associated death, which was supported by more stable hemodynamic, metabolic and inflammatory statuses in patients receiving intraoperative hemoadsorption [13]. In the current study, the incidence of postoperative sepsis was comparable between the two groups. However, sepsis-related mortality was significantly reduced in patients with intraoperative hemoadsorption. This observation was supported by faster recovery of cardiopulmonary status and SOFA scores in the hemoadsorption as compared to the control group. These data suggest that hemoadsorption therapy attenuates the severity of sepsis in high-risk patients, thus preventing development of septic shock leading to death. Overall in-hospital mortality was high and comparable between the two groups. Among the in-hospital deaths, five patients died due to secondary infections, three in the hemoadsorption group and two in the control group. Secondary infections after cardiac surgery occur due to the biphasic immune response, in which the second phase is dominated by an anti-inflammatory reaction [18, 19]. Additionally, in the hemoadsorption group two patients died unexpectedly due to in-hospital cardiac arrest after a hospital transfer where they had been in a stable condition.

An interesting observation was that respiratory failure occurred significantly less often in the hemoadsorption group than in the control group. These data are consistent with previous studies showing that patients with infective endocarditis are at increased risk for developing postoperative respiratory failure, and postoperative respiratory failure is an important predictive factor of morbidity and mortality [20, 21].

Hemoadsorption during cardiopulmonary bypass is currently a frequent theme of clinical research in cardiac surgery. Hemoadsorption therapy has been shown to reduce circulating cytokines at various levels [22–25]. The variability in the results could be explained by the study design and sample size, patient characteristics, and timing and duration of the therapy. Although patients with infective endocarditis have higher concentrations of cytokines [5, 26], evaluation of intraoperative hemoadsorption therapy in patients with infective endocarditis is less studied in the literature. In these two retrospective studies, intraoperative hemoadsorption seemed to reduce the severity of postoperative sepsis and sepsis-associated mortality by stabilizing hemodynamics and inflammatory response [13, 27]. It should be kept in mind that removal of circulating cytokines only has an impact on inflammatory response. Although postoperative sepsis is an important cause of surgical mortality, mortality by other causes cannot be prevented by hemoadsorption therapy.

Currently, two randomized controlled clinical trials are currently ongoing to evaluate the effects of intraoperative hemoadsorption therapy in patients with infective endocarditis [28, 29]. The primary endpoints of these two randomized trials are focused on Sequential Organ Failure Assessment and monocytic human leukocyte antigen-DR expression. In our study, the focus was on the clinical effects of intraoperative hemoadsorption in high-risk patients with infective endocarditis. Although this study is not a randomized clinical trial, the two groups of patients were comparable after propensity score matching. Nevertheless, bias cannot be completely excluded. Further, the retrospective design precluded routine evaluation of the whole cytokine panel, such as TNFα, IL-6 or IL-1β. Nonetheless, this is to the best of our knowledge the largest and first study to evaluate intraoperative hemoadsorption in a high-risk cohort undergoing cardiac surgery for infective endocarditis thus far. Regarding high-risk definition, the cutoff point for determining high-risk patients was set at a EuroSCORE II greater than 8% as the EuroSCORE II overestimates the operative mortality in cardiac surgical patients [30, 31]. In order to compensate for this overestimation, we defined a high-risk patients as higher than 6% as in other studies.

In conclusion, intraoperative hemoadsorption seems to attenuate the severity of postoperative sepsis and reduce sepsis-associated mortality in high-risk patients undergoing surgical therapy for infective endocarditis. Future studies are mandatory to confirm the current results in a larger population and to explore the underlying mechanisms behind these effects.

## Supporting information

S1 Data (XLSX)
